# Bayesian Network Modeling of Environmental, Social, and Behavioral Determinants of Cardiovascular Disease Risk

**DOI:** 10.3390/ijerph22101551

**Published:** 2025-10-12

**Authors:** Hope Nyavor, Emmanuel Obeng-Gyasi

**Affiliations:** 1 Department of Built Environment, North Carolina A&T State University, Greensboro, NC 27411, USA; 2 Environmental Health and Disease Laboratory, North Carolina A&T State University, Greensboro, NC 27411, USA

**Keywords:** PFAS, metals, inflammation, socioeconomic status, Bayesian network, cardiovascular disease (CVD), allostatic load, C-reactive protein (CRP), grow–shrink, hill-climbing

## Abstract

Background: Cardiovascular disease (CVD) is the leading global cause of death and is shaped by interacting biological, environmental, lifestyle, and social factors. Traditional models often treat risk factors in isolation and may miss dependencies among exposures and biomarkers. Objective: To map interdependencies among environmental, social, behavioral, and biological predictors of CVD risk using Bayesian network models. Methods: A cross-sectional analysis was conducted using NHANES 2017–2018 data. After complete-case procedures, the analytic sample included 601 adults and 22 variables: outcomes (systolic/diastolic blood pressure, total/LDL/HDL cholesterol, triglycerides) and predictors (BMI, C-reactive protein (CRP), allostatic load, Dietary Inflammatory Index, income, education, age, gender, race, smoking, alcohol, and serum lead, cadmium, mercury, and PFOA). Spearman’s correlations summarized pairwise associations. Bayesian networks were learned with two approaches: Grow–Shrink (constraint-based) and Hill-Climbing (score-based, Bayesian Gaussian equivalent score). Network size metrics included number of nodes, directed edges, average neighborhood size, and Markov blanket size. Results: Correlation screening reproduced expected patterns, including very high systolic–diastolic concordance (*p* ≈ 1.00), strong LDL–total cholesterol correlation (*p* = 0.90), inverse HDL–triglycerides association, and positive BMI–CRP association. The final Hill-Climbing network contained 22 nodes and 44 directed edges, with an average neighborhood size of ~4 and an average Markov blanket size of ~6.1, indicating multiple indirect dependencies. Across both learning algorithms, BMI, CRP, and allostatic load emerged as central nodes. Environmental toxicants (lead, cadmium, mercury, PFOS, PFOA) showed connections to sociodemographic variables (income, education, race) and to inflammatory and lipid markers, suggesting patterned exposure linked to socioeconomic position. Diet and stress measures were positioned upstream of blood pressure and triglycerides in the score-based model, consistent with stress-inflammation–metabolic pathways. Agreement across algorithms on key hubs (BMI, CRP, allostatic load) supported network robustness for central structures. Conclusions: Bayesian network modeling identified interconnected pathways linking obesity, systemic inflammation, chronic stress, and environmental toxicant burden with cardiovascular risk indicators. Findings are consistent with the view that biological dysregulation is linked with CVD and environmental or social stresses.

## 1. Introduction

Cardiovascular disease (CVD) remains the leading cause of morbidity and mortality worldwide, responsible for approximately one-third of global deaths annually [1]. The burden of CVD is not only a significant public health challenge but also a major contributor to healthcare costs. According to the Global Burden of Disease Study, CVD accounted for over 18.6 million deaths in 2019, underscoring its pervasive impact across both developed and developing nations [2]. The high prevalence of CVD risk factors such as hypertension, hyperlipidemia, and diabetes is influenced by environmental, behavioral, and socioeconomic determinants. These risk factors are not isolated but are strongly influenced by a combination of environmental, behavioral, and socioeconomic factors. For example, poor eating habits, lack of exercise, and exposure to harmful substances in the environment are major contributors to CVD risk [3].

CVD is not solely attributable to individual behavioral choices but rather arises from a complex interplay of multiple determinants, including dietary patterns, exposure to environmental contaminants, chronic psychosocial stress, and broader social and economic conditions. [4]. Additionally, factors such as income gaps, education levels, and access to healthcare services also play a significant role in determining who faces the highest risks and how effectively those risks are addressed. Understanding how these factors interact is crucial for developing effective strategies to prevent and manage CVD.

Long-term exposure to heavy metals like lead, cadmium, and mercury is becoming more widely acknowledged as a significant cause of cardiovascular disorders, which include atherosclerosis, high blood pressure, and altered blood vessel function [5]. The two primary processes by which these metals damage the heart and blood arteries are oxidative stress and chronic inflammation [6]. While air pollution’s role in heart disease is well-documented, growing evidence now links metals such as lead, cadmium, and arsenic to cardiovascular risks worldwide [7]. People encounter these metals daily through contaminated air, water, soil, food, and industrial activities. For example, even low-level lead exposure can damage blood vessels and raise blood pressure, while cadmium and mercury disrupt the delicate lining of arteries, accelerating plaque buildup [8]. Therefore, actively minimizing exposure to these metals should be included as a key part of broader efforts to prevent CVD [9].

Per- and Polyfluoroalkyl Substances, or PFAS, often referred to as “forever chemicals,” are increasingly recognized as harmful environmental pollutants. Commonly found in products like non-stick cookware, waterproof fabrics, and firefighting foams, these chemicals interfere with hormone systems and have been linked to unhealthy cholesterol levels and chronic inflammation in blood vessels, both of which increase the risk of heart disease [10]. The persistence of PFAS in the environment and their bioaccumulation in human tissues make them a public health concern.

The impact of environmental toxicants on cardiovascular health can be either minimized or exacerbated by the quality of one’s diet, which encompasses nutrient intake and the consumption of processed foods. Inflammation and oxidative stress caused by environmental exposures can be lessened with a diet high in fruits, vegetables, healthy grains, and lean meats. On the other hand, diets heavy in processed foods, added sugars, and saturated fats can raise the risk of CVD and speed up the negative effects of toxicants [11]. For example, omega-3 fatty acids from fish reduce the inflammation linked to PFAS exposure, while antioxidants in plant-based meals may reduce the oxidative stress produced by heavy metals [12]. These results underscore the importance of promoting healthy eating habits as part of measures to prevent CVD.

Allostatic load, a cumulative measure of physiological wear and tear resulting from chronic stress exposure, is associated with elevated systemic inflammation and an increased risk of cardiovascular disease [13]. Individuals experiencing persistent social adversity, such as poverty, discrimination, or other structural stressors, are disproportionately burdened by heightened allostatic load, thereby facing greater cardiovascular risk [14]. Mitigating these health issues requires addressing upstream social determinants of stress and expanding access to comprehensive health services.

Income and educational attainment have a significant influence on access to healthcare services, nutritional quality, and the likelihood of exposure to environmental hazards. Individuals with lower socioeconomic status (SES) are disproportionately likely to reside in areas characterized by elevated air pollution, contaminated water sources, and limited availability of nutritious food options. These adverse environmental and social conditions contribute to an increased risk of CVD [15]. Additionally, lower education levels are associated with reduced health literacy, which can hinder the adoption of preventive behaviors and adherence to medical treatments [16]. These differences underscore the need for targeted measures to improve healthcare access and mitigate environmental disparities [17].

Socioeconomic factors can also magnify susceptibility to environmental exposures. For example, individuals with limited financial resources may have fewer opportunities to mitigate the effects of environmental toxicants, such as by purchasing air purifiers or relocating to less polluted areas. Similarly, chronic stress from socioeconomic adversity can amplify the cardiovascular effects of environmental exposures, creating a synergistic risk for CVD [18]. Addressing these interconnected issues requires a holistic approach that includes community-based programs, legislative reforms, and equitable access to healthcare.

However, much of the cardiovascular literature still relies on models that assume linear relationships, treat exposures one at a time, and do not represent uncertainty transparently. This leaves key questions unanswered when exposures co-occur, interact, or operate through indirect pathways. Bayesian networks (BN) address this gap by allowing non-linear and conditional relationships, by explicitly propagating uncertainty across the graph, and by modeling the joint effects of multiple variables, including co-exposures, upstream social conditions, and potential measurement error. In the context of environmental and social determinants of cardiovascular risk, this approach is especially significant because it can map how metals and PFAS are linked with diet, stress biology (e.g., allostatic load), and blood-pressure profiles, while distinguishing variables on direct versus indirect paths.

A BN is like a smart flowchart that maps out how different factors (e.g., age, diet, blood pressure) influence one another and the risk of a disease (such as heart disease). It uses probabilities to show cause-and-effect relationships [19]. For example, if someone smokes (factor A), how much does that increase their risk of high blood pressure (factor B), and how does that then affect their heart disease risk [20]. BNs are powerful tools for understanding complex relationships between different factors. Their ability to distinguish between the direct and indirect effects of factors is one of their primary advantages. For instance, a bad diet may generate high cholesterol first, making it an indirect cause of heart disease, even though high cholesterol may directly contribute to it [21]. By separating these factors, Bayesian networks enable the identification of what truly causes health concerns. Their capacity to integrate current knowledge into model building is another crucial aspect. To increase accuracy, they can incorporate prior research, professional judgments, or clinical results, rather than relying solely on new data. Because of this, they are especially helpful when making medical decisions, where past information is crucial [22].

The potential of BNs to identify modifiable risk factors in aspects that can be altered to reduce disease risk is a significant benefit in CVD research [23]. For instance, if it is discovered that education and income level affect access to healthcare and nutritious food, policymakers can utilize this data to create initiatives that increase access to healthcare and wholesome food options in lower-income areas [24].

CVD arises from a constellation of interacting risk factors, ranging from biological and behavioral to environmental and social, which disproportionately affect vulnerable populations [25]. Environmental pollutants such as toxic metals and PFAS have been linked to endothelial dysfunction, oxidative stress, inflammation, and metabolic disruption, all of which are critical pathways in the pathogenesis of CVD [26]. These toxicants may also contribute to dysregulation of the neuroendocrine and immune systems, amplifying chronic stress responses and exacerbating cardiovascular risk.

Dietary behaviors, which play a crucial role in cardiovascular health, are closely linked to environmental exposures and chronic psychosocial stress. These factors are further shaped by socioeconomic status (SES), including income and education, which influence access to healthcare, nutritious food, and safe living environments [27]. Psychobiological processes linked to systemic inflammation and cardiovascular dysregulation are activated by chronic stress, which is frequently made worse by socioeconomic hardship [28]. Individuals with lower SES are more likely to experience cumulative disadvantage—such as exposure to air and water pollution, food insecurity, and persistent stressors—that jointly elevate the risk of CVD [29].

Structural factors such as economic disadvantages and unequal access to resources further compound environmental health disparities. The convergence of social and environmental stressors contributes to a disproportionate burden of CVD in certain populations [30]. According to the social determinants of health framework, addressing both environmental injustice and socioeconomic inequities is crucial for reducing disparities in cardiovascular outcomes [31]. Given this complex interplay, it is crucial to investigate how diet, environmental exposures (including metals and PFAS), psychosocial stress, and socioeconomic indicators collectively impact cardiovascular risk. The present study adopts an integrative approach to characterize these multidimensional relationships, setting the stage for our analytic methods.

## 2. Materials and Methods

### 2.1. Study Design

This study utilized publicly available data from the National Health and Nutrition Examination Survey (NHANES) 2017–2018, conducted by the U.S. Centers for Disease Control and Prevention (CDC). The National Center for Health Statistics Ethics Review Board reviewed and approved NHANES protocols to ensure that studies involving human participants protected their rights and welfare and complied with U.S. federal regulations. All NHANES participants provided written informed consent. NHANES employs a complex, multistage probability sampling design to obtain a nationally representative sample of the non-institutionalized civilian population across all 50 U.S. states. The survey integrates interviews, physical examinations, and laboratory tests to assess the health and nutritional status of participants.

To ensure transparency regarding population representation, we first generated benchmark descriptive estimates and correlations using NHANES weights and design factors. These weighted results were then compared with the unweighted data used in the Bayesian network analysis. Because few tools are currently available to fully integrate complex survey weighting into network learning, the unweighted approach was considered appropriate. This decision was further supported by the consistency in the relative magnitudes and directions of key relationships across both weighted and unweighted non-Bayesian analyses.

### 2.2. Database and Sample Size

To ensure the quality and completeness of the analytical dataset, we conducted data cleaning procedures and excluded observations with missing values for any of the 22 selected variables representing exposures, outcomes, and covariates. This complete case approach resulted in a final analytic sample of 601 participants with no missing data. This refined dataset was used for all subsequent statistical analyses. The variables include six outcome variables related to cardiovascular health and 16 predictor variables representing biological, environmental, lifestyle, and socioeconomic factors. AL was operationalized as a cumulative index of physiological dysfunction across the cardiovascular (SBP, DBP, triglycerides, high-density lipoprotein (HDL) cholesterol, total cholesterol), inflammatory (CRP), and metabolic (BMI, hemoglobin A1C, albumin, creatinine clearance) systems. For each biomarker, participants received a score of 1 if values were at or above the 75th percentile (SBP, DBP, triglycerides, total cholesterol, BMI, CRP, hemoglobin A1C) or at or below the 25th percentile (HDL cholesterol, albumin, creatinine clearance), with high-risk cut points determined from the sample distribution. Scores were summed to generate an AL index ranging from 0 to 10, with higher values indicating greater multisystem physiological dysregulation.

A complete-case approach was used for the network analysis. This approach assumes that data are missing completely at random; if that assumption is not met, estimates and learned edges can be biased and the analytic sample may be less representative of the target population. To gauge potential selection bias, characteristics available prior to exclusion (age, sex, race/ethnicity, education, and income) were compared between included and excluded participants; differences were small to moderate, so results are presented as associations within this analyzed subset. Future work will evaluate multiple imputations and survey-weighted learning to improve inference.

The critical variables for the study are described in Table 1.

#### 2.2.1. Measurement of Key Variables

We analysed a set of biomarkers and covariates drawn from the 2017–2018 (NHANES). All measurements were obtained under standardized NHANES protocols or derived from those measurements.

##### Cardiovascular Biomarkers (Outcome Variables)

Blood pressure (systolic and diastolic) was measured in the Mobile Examination Center (MEC) by trained technicians. After a 5 min seated rest, three consecutive readings were taken on the right arm using a cuff matched to mid-upper arm circumference; a fourth reading was taken if needed. Examiners followed strict calibration and quality control procedures. The mean of valid measurements was used in analysis. Serum total cholesterol was measured enzymatically on a Roche Cobas analyzer: cholesterol esters are hydrolysed and oxidised, and the hydrogen peroxide produced reacts in a peroxidase-coupled assay read at ~500 nm. High-density lipoprotein cholesterol (HDL-C) was quantified with a homogeneous direct assay that blocks apoB-containing lipoproteins and enzymatically measures cholesterol in HDL. Triglycerides were assayed enzymatically by hydrolysing triglycerides to glycerol, which is then phosphorylated and oxidised; the resulting hydrogen peroxide generates a chromogenic product measured spectrophotometrically. Low-density lipoprotein cholesterol (LDL-C) was calculated via the Friedewald equation: LDL-C = total cholesterol − HDL-C − (triglycerides/5). High-sensitivity C-reactive protein (hs-CRP) was measured using an immunoturbidimetric assay in which serum CRP forms immune complexes with latex-bound antibodies; turbidity proportional to CRP concentration was measured at 546 nm.

##### Health Predictors

Body mass index (BMI) was derived from measured weight and standing height taken by certified technicians in the MEC. Weight and height were recorded to the nearest 0.1 kg and 0.1 cm, respectively, following the NHANES Anthropometry Procedures Manual, and BMI was calculated as weight/height^2^ (kg/m^2^). To summarise multisystem physiological stress, we constructed an allostatic load score using seven biomarkers (systolic and diastolic blood pressure, BMI, total cholesterol, LDL-C, triglycerides, and CRP, with HDL-C reverse-coded). Each biomarker contributed one point if it fell in the sex-specific 75th percentile (or 25th percentile for HDL-C), yielding a score from 0 to 8. Dietary inflammatory index (DII) scores were calculated from 24 h dietary recall data. Twenty-eight nutrients were assigned +1 or −1 depending on whether they were associated with higher pro-inflammatory cytokines (CRP, IL-1β, IL-6, TNF-α) or anti-inflammatory cytokines (IL-4, IL-10); each component was weighted by its literature-derived inflammatory effect and the participant’s standardized intake, then summed to yield the DII. Higher DII values indicate more pro-inflammatory diets. C-reactive protein (CRP) served both as an outcome (hs-CRP) and as a component of allostatic load.

##### Environmental Exposure Biomarkers

Perfluorooctanoic acid (PFOA) and perfluorooctane sulfonic acid (PFOS) were measured in serum using automated online solid-phase extraction coupled with high-performance liquid chromatography–tandem mass spectrometry. The method separates PFAS compounds chromatographically and detects them by tandem MS; it quantifies linear and branched isomers of PFOS and PFOA and captures newer PFAS such as GenX and ADONA. Blood lead, cadmium, and total mercury were quantified by inductively coupled plasma–dynamic reaction cell mass spectrometry. Whole blood was vortexed, then diluted (1:1:48) with a solution containing tetramethylammonium hydroxide, Triton X-100, ammonium pyrrolidine dithiocarbamate and ethanol; internal standards (rhodium, iridium and tellurium) were added. Diluted samples were nebulised into an argon plasma, and ions passed through a dynamic reaction cell—pressurised with methane or oxygen to eliminate polyatomic interferences—before quadrupole mass analysis. This method allows simultaneous determination of cadmium, lead, mercury, manganese and selenium.

##### Lifestyle, Socioeconomic and Demographic Covariates

Smoking status and alcohol consumption were obtained from interviewer-administered questionnaires. Participants reported lifetime and current smoking, permitting classification as never, former or current smokers, and reported frequency of alcoholic beverage consumption, which was categorised as non-drinker, moderate or heavy drinker. Socioeconomic variables included education (self-reported highest grade completed) and the family poverty-income ratio (PIR), the ratio of family income to the federal poverty threshold. Demographic covariates comprised age (in years), sex (male or female, based on self-reported sex at birth) and race/ethnicity (Non-Hispanic White, Non-Hispanic Black, Mexican American, Other Hispanic or Other Race/multiracial), all recorded via the NHANES Demographic Questionnaire.

### 2.3. Inclusion and Exclusion Criteria

To enhance the reliability and validity of the analysis, specific inclusion and exclusion criteria were applied to the NHANES 2017–2018 dataset. Individuals were included if they were aged 20 years or older, as cardiovascular risk factors typically accumulate and manifest over time. In addition, participants were required to have complete data on key cardiovascular health indicators, including blood pressure and cholesterol levels.

Exclusion criteria were also implemented to avoid introducing bias and to maintain data quality. Participants with missing values for critical cardiovascular measurements were excluded from the analysis, as incomplete data could compromise model accuracy. Furthermore, observations with extreme outliers in blood pressure or cholesterol values—likely due to measurement error or data entry inaccuracies—were removed to prevent distortion of statistical estimates.

By systematically applying these criteria, the final analytic sample reflected a well-defined population suitable for assessing associations between environmental exposures and cardiovascular health outcomes. Because participants with any missing values on the selected variables were excluded, the final sample reflects individuals with more complete measurements. Although this improves internal consistency for modeling, it can reduce external validity if missingness relates to sociodemographic factors or health status. Accordingly, findings are interpreted as patterns within the analyzed sample, and potential selection bias is considered in the Limitations

### 2.4. Statistical Techniques

#### 2.4.1. Descriptive Statistics

Descriptive statistics were calculated to summarize the central tendencies and distributions of key health-related variables in the study sample. Measures including the minimum, 25th percentile, median, mean, 75th percentile, and maximum values were reported for variables such as blood pressure (systolic and diastolic), cholesterol levels (LDL, HDL, and total), BMI, allostatic load, and the DII. These statistics provided a foundational overview of participants’ cardiovascular and metabolic health status.

#### 2.4.2. Spearman Correlation Analysis

We applied the Spearman rank correlation to assess the strength and direction of monotonic associations between variables related to cardiovascular health. This method is well-suited for situations where the relationship between variables is not necessarily linear but still follows a consistent trend, either increasing or decreasing. Unlike Pearson’s correlation, Spearman’s approach is non-parametric and relies on the rankings of data points rather than their raw values, making it more robust to the influence of outliers. This characteristic is particularly advantageous when the dataset includes extreme values. The Spearman correlation coefficient is defined as follows:

$$r_{s}=1-\frac{6\sum d_{i}^{2}}{n(n^{2}-1)}$$

In this formula, d_i_ represents the difference between the ranks of each paired observation, and n stands for the total number of observations. The Spearman correlation coefficient, denoted as r_s_, ranges from −1 to +1. A positive r_s_ value suggests that as one variable increases, the other tends to increase as well. Conversely, a negative r_s_ indicates that one variable decreases as the other increases. When r_s_ = 0, it implies there is no consistent relationship between the two variables. Statistical significance for all associations was evaluated at α = 0.05 (two-sided). Correlations were considered statistically significant when *p* < 0.05.

#### 2.4.3. Bayesian Network Model

A Bayesian Network (BN) is a type of probabilistic graphical model used to represent relationships between a set of random variables. It is particularly useful in decision-making scenarios where uncertainty is involved. The key strength of Bayesian Networks lies in their ability to model conditional dependencies between variables, making them valuable in fields such as medicine and genetics. Currently, widely used BN structure-learning implementations do not natively incorporate complex survey weights. Accordingly, BN models were fit to the complete-case analytic set without design weights. To assess robustness, design-consistent weighted descriptive and correlation results were compared with BN-suggested dependencies; the primary directions of connection were consistent.

Structure of a Bayesian Network

A Bayesian Network is mathematically represented as B=(G,Θ), where

- B represents the Bayesian Network.
- G is a Directed Acyclic Graph (DAG), where nodes (X1, X2, …, Xn) represent random variables, and directed edges between them signify dependencies.
- Θ are the set parameters that define the conditional probability distributions governing these variables.

Mathematical Representation of a Bayesian Network

A Bayesian Network encodes a joint probability distribution (JPD) over a set of variables V. This is defined by:

$$P\left(X_{1},X_{2},\ldots X_{n}\prod_{i=1}^{n}P\left(\left.X_{i}\right|Parents\left(X_{i}\right)\right.\right)$$

This equation states that the probability of a variable $X_{i}$ depends only on its parent nodes in the network thereby simplifying complex probability computations. Bayesian Networks have become a fundamental tool in fields requiring probabilistic modeling, providing a structured and efficient way to represent relationships among uncertain variables. For the constraint-based Grow–Shrink learning, conditional-independence tests used a 0.05 significance threshold; edges retained reflect dependencies that were not rejected at α = 0.05.

## 3. Results

### 3.1. Descriptive Analysis of Cardiovascular and Metabolic Health Indicators

The descriptive statistics, as presented in Table 2, provide a clear picture of the participants’ cardiovascular health. Both diastolic and systolic blood pressure levels fall within the expected population ranges. When looking at cholesterol levels, LDL cholesterol has a median value of 106 mg/dL, but levels above 130 mg/dL are considered a risk factor for heart disease. On the other hand, HDL cholesterol, known as “good” cholesterol, has a median of 51 mg/dL, with values above 60 mg/dL being ideal for heart health. Additionally, the BMI distribution suggests that most participants fall within the overweight to obese range, which could contribute to increased cardiovascular risk.

### 3.2. Spearman Correlation Results

**Figure 1:**
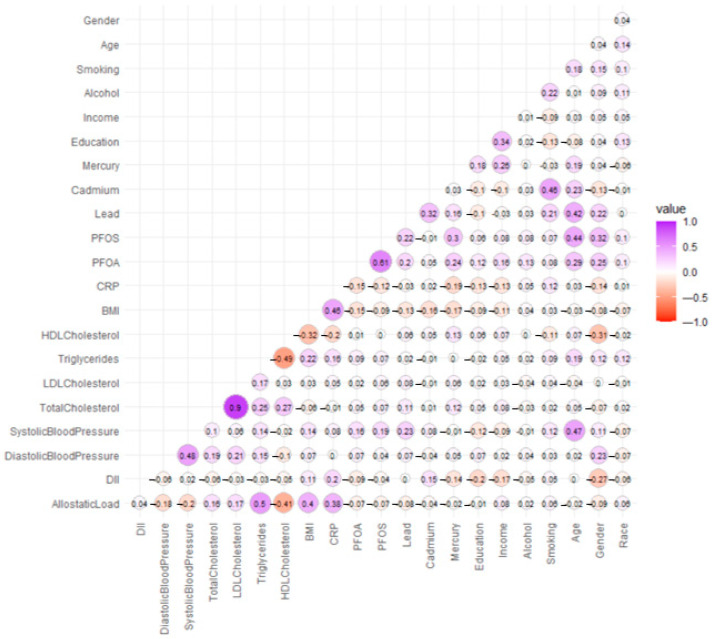
Spearman correlation matrix.

The Spearman correlation matrix was used to evaluate the strength and direction of monotonic associations among study variables, providing insight into the interrelationships between exposures, outcomes, and covariates (Figure 1). The color scale ranges from red (strong positive correlation, close to 1) to blue (strong negative correlation, close to −1), with white indicating weak or no correlation. As expected, systolic and diastolic blood pressure are highly correlated (1.00), meaning they tend to increase or decrease together. Similarly, LDL cholesterol and total cholesterol exhibit a strong positive correlation (r = 0.90), which is expected since LDL is a major component of total cholesterol. BMI and CRP also have a positive correlation, suggesting that a higher BMI is associated with increased inflammation. On the other hand, HDL cholesterol (good cholesterol) has a negative correlation with triglycerides and allostatic load, meaning that as HDL increases, these risk factors decrease. Environmental exposures such as lead, cadmium, and mercury show moderate correlations with other risk factors, indicating possible links between toxic metal exposure and cardiovascular health.

This analysis highlights key interactions between biological, lifestyle, and environmental factors, offering deeper insights into cardiovascular disease risks.

### 3.3. Bayesian Network Learning Using the Grow-Shrink Algorithm

To elucidate the conditional dependencies among variables in the dataset, the Grow-Shrink (GS) algorithm was employed (Table 3). This algorithm is a constraint-based structure learning method within the Bayesian network framework that identifies the Markov blanket of each variable by iteratively adding and removing variables based on conditional independence tests. The analysis yielded an average Markov blanket size of 5.00, indicating that, on average, each variable is conditionally dependent on approximately five others. The average neighborhood size was 4.00, suggesting that each variable is directly connected to about four others in the learned network structure. Overall, the algorithm identified 44 direct connections (edges) between variables, mapping out a structure of relationships that may impact cardiovascular risk.

### 3.4. Bayesian Network Learning Using the Hill-Climbing Algorithm

To refine the structure of the Bayesian Network, the Hill-Climbing (HC) algorithm was used. It is a method that builds the network by incrementally improving connections based on a scoring system. Unlike constraint-based approaches, which rely on statistical tests to determine relationships, the HC algorithm searches for the best-fitting structure by adding, removing, or reversing connections between variables. This approach allows for a more flexible and data-driven refinement of the network. The final network consisted of 22 nodes, representing the key variables in the dataset, and 44 directed arcs, which indicate the relationships between them. On average, each variable had 6.09 direct influences (Markov blanket size) and was connected to about four other variables (neighborhood size). Additionally, each variable influenced approximately two other variables (branching factor), illustrating the network’s complexity. The model was evaluated using the Bayesian Gaussian Equivalent (BGe) score, a function specifically designed for continuous data, ensuring that the network effectively captured relationships between cardiovascular risk factors. A detailed summary of the Bayesian network parameters, including scoring metrics, prior assumptions, and model optimization details, is presented in Table 4.

### 3.5. Cardiovascular Network Structure from Grow-Shrink Bayesian Model

**Figure 2:**
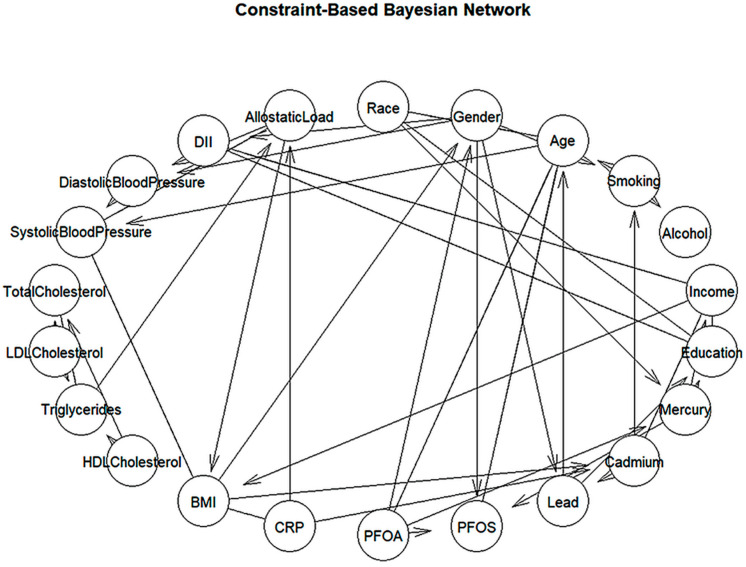
Constraint-Based Bayesian Network constructed using the Grow-Shrink (GS) algorithm with circular layout.

To uncover complex relationships among cardiovascular, environmental, behavioral, and sociodemographic factors, a Constraint-Based Bayesian Network (CBBN) was constructed (Figure 2). This data-driven approach tests for conditional independence between variables and maps only the most significant associations, allowing for the identification of both direct and indirect influences without relying on a predefined structure. The resulting network highlights key variables such as BMI and CRP as central hubs, linking dietary inflammation, lipid profiles, and systemic inflammation, highlighting their critical role in CVD risk. It also reveals strong connections between environmental toxicants (e.g., lead, PFOS, PFOA) and sociodemographic factors, such as age, gender, income, and education, suggesting that exposure to harmful substances is shaped by both structural and social factors.

Moreover, the model underscores the influence of diet and chronic stress on hypertension. As demonstrated by the associations between the DII, allostatic load, and blood pressure, lifestyle-related factors significantly contribute to cardiovascular risk. These findings support the need for interventions focused on stress reduction and anti-inflammatory dietary habits to help prevent hypertension and related conditions.

Overall, the CBBN approach provides a comprehensive understanding of how biological, environmental, and social factors interact to shape cardiovascular health. These insights can guide the development of targeted, multi-level public health strategies, particularly for vulnerable populations disproportionately affected by environmental exposures and chronic stress.

### 3.6. Cardiovascular Network Structure from Hill-Climbing Bayesian Model

**Figure 3:**
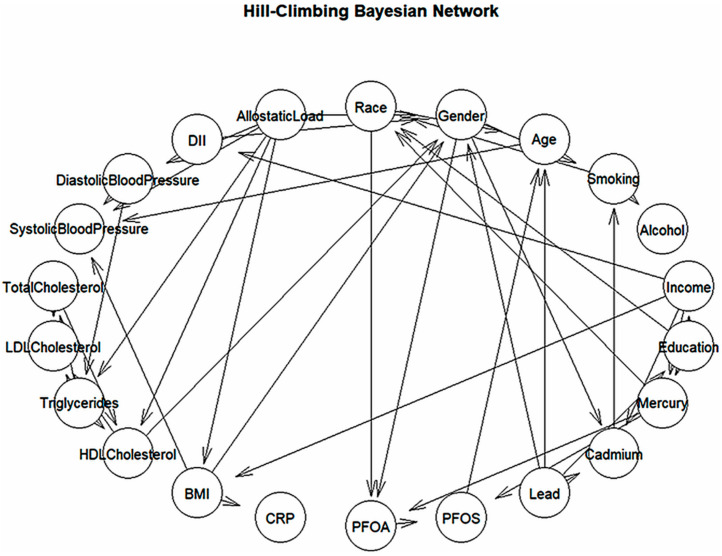
Hill-Climbing Bayesian Network using a score-based approach with circular layout.

The Hill-Climbing Bayesian Network (HC-BN) provides a structured representation of the relationships between different factors influencing cardiovascular health (Figure 3). The network is composed of two primary types of variables: predictor variables (causes) and outcome variables (effects). Predictor variables include diet, environmental exposures, stress, socioeconomic factors, and lifestyle choices, all of which have the potential to influence cardiovascular outcomes. Outcome variables represent key indicators of cardiovascular health, including blood pressure, cholesterol levels, and triglycerides. The arrows (edges) in the network illustrate direct dependencies, meaning that if one variable points to another, it suggests an influence or potential causal relationship between them. Unlike constraint-based models, the HC-BN refines these relationships by maximizing a scoring function, allowing for the identification of more complex interactions. The Bayesian Network also offers key insights into broader social and environmental determinants of cardiovascular health. The model highlights that obesity and inflammation are central risk factors, with BMI and CRP affecting cholesterol, triglycerides, and overall cardiovascular health. This suggests that targeting obesity and reducing systemic inflammation through diet, physical activity, and medical interventions could significantly lower heart disease risk. Additionally, the network highlights that exposure to environmental pollutants is linked to socioeconomic disparities, with Lead, Mercury, and Cadmium disproportionately affecting lower-income and less educated populations.

### 3.7. Hierarchical Visualization of Constraint-Based Cardiovascular Network

**Figure 4:**
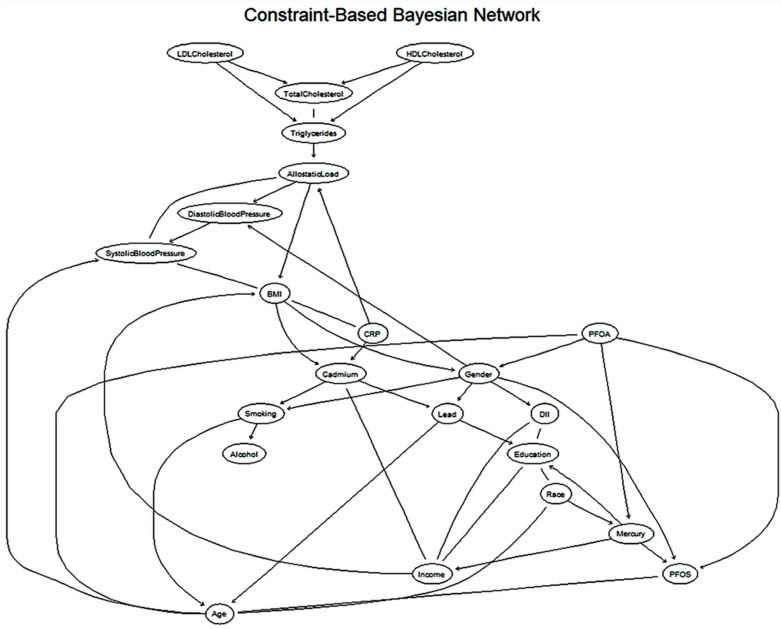
Constraint-Based Bayesian Network using the Grow-Shrink algorithm with hierarchical (layered) layout.

The results of the constraint-based Bayesian network analysis are presented in Figure 4, illustrating the conditional dependencies among key predictor variables and cardiovascular health outcomes. Directed edges represent potential direct influences between variables. Notably, both LDL and HDL are upstream of total cholesterol, which in turn is linked to triglyceride levels, indicating a cascade of lipid-related influences.

BMI emerges as a central node in the network, directly associated with systolic and diastolic blood pressure, allostatic load, CRP, and cadmium levels. BMI itself is influenced by behavioral and demographic factors, including smoking status, age, and inflammatory status (via CRP), highlighting its multifactorial nature.

Several environmental toxicants, such as lead, cadmium, mercury, PFOS, and PFOA, are also integrated into the network. Lead and cadmium levels are conditionally dependent on gender and smoking, while mercury is associated with race, PFOS, and education level.

### 3.8. Hierarchical Visualization of Score-Based Cardiovascular Network

**Figure 5:**
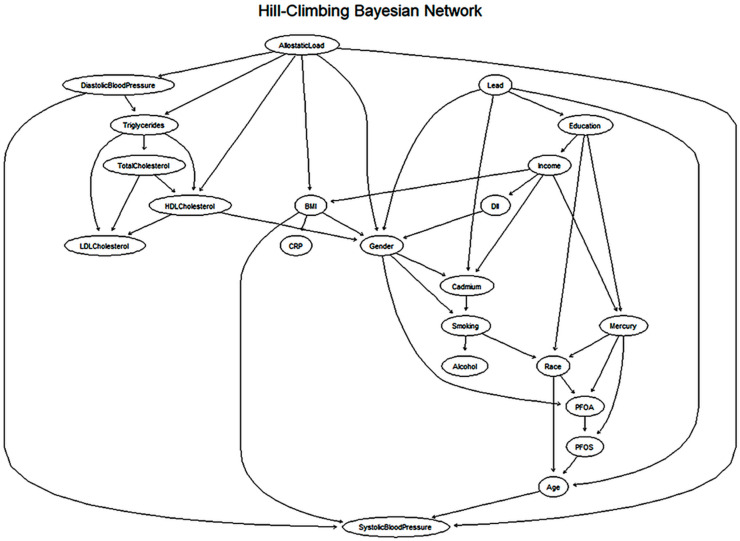
Hill-Climbing Bayesian Network using a score-based approach with hierarchical layout.

Figure 5 presents a Hill-Climbing Bayesian Network that maps conditional dependencies among health, behavioral, environmental, and sociodemographic variables. The model identifies allostatic load as a key upstream factor influencing blood pressure, triglycerides, and BMI, underscoring the role of chronic stress in cardiometabolic health. BMI in turn affects both CRP and HDL cholesterol, reflecting pathways linking body weight to inflammation and lipid metabolism.

Environmental toxicants lead, cadmium, PFOA, PFOS, and mercury are closely tied to behavioral and demographic variables such as smoking, alcohol use, income, education, gender, and age, indicating that exposure risk is shaped by lifestyle and structural factors. This network provides a more nuanced view of how interconnected stress, environment, and social determinants contribute to cardiovascular disease risk.

## 4. Discussion

### 4.1. Overview of Key Findings

The analysis revealed several important trends in cardiovascular risk factors. BMI was generally high among participants, and CRP levels a marker of inflammation were also elevated. Notably, BMI showed a positive correlation with CRP, indicating that individuals with higher BMI tended to have higher systemic inflammation. This finding is biologically plausible: obesity is known to promote chronic, low-grade inflammation through the release of cytokines from adipose tissue, leading to elevated CRP levels. In fact, meta-analyses and population studies consistently report that higher BMI is associated with higher CRP, supporting the link observed in our data [32]. This suggests that excess body weight contributes to an inflammatory state that can elevate cardiovascular risk. Lipid correlations in the data align with known patterns. We found that HDL cholesterol was inversely correlated with triglycerides [33]. It reinforces HDL’s role as a protective factor: higher HDL levels are generally associated with lower cardiovascular risk [34]. These summary patterns support known lipid and inflammation dynamics and are therefore referenced only once here, with detailed interpretation reserved for the network results.

### 4.2. Interpretation of Descriptive and Correlational Results

Descriptive and correlational results indicated a coherent cardiometabolic pattern in the analytic sample. Higher BMI was associated with higher CRP, and HDL was inversely associated with triglycerides, consistent with well-described metabolic relations observed in population studies. Moderate positive correlations between heavy metals (for example, lead and cadmium) and both blood pressure and CRP further suggested environmentally related inflammatory and vascular pathways. Taken together, these patterns provided face validity for proceeding to Bayesian network modeling to distinguish direct from indirect associations and to situate social and environmental factors alongside biological indicators. For instance, individuals with higher lead or cadmium levels tend to had higher blood pressure or CRP [35]. These associations aligned with evidence linking toxic metal exposure to hypertension and inflammation, including reports that even low-level lead exposure elevates blood pressure and that cadmium exposure is associated with greater hypertension risk [36]. Such metals induce oxidative stress and vascular inflammation, which may explain the correlation with CRP (an inflammation marker). Overall, the descriptive statistics and correlation analysis paint a coherent picture: traditional risk factors, such as obesity, dyslipidemia, and hypertension, are interrelated and influenced by both biological processes (inflammation and metabolism) and external exposures. These patterns are consistent with known pathophysiology and set the stage for more complex relationship modeling.

### 4.3. Network Analysis Insights

#### 4.3.1. Constraint-Based (Grow-Shrink) Network

The constraint-based Bayesian network (learned via the Grow-Shrink algorithm) provided a simplified map of the strongest direct dependencies among variables. A striking insight from this model was the prominence of BMI and CRP as central nodes in the network. Both appeared as hubs connecting to multiple other factors, indicating their pivotal role in the web of cardiometabolic relationships. For instance, BMI was directly linked with blood pressure, lipid measures, and CRP, while CRP was connected with factors like the DII and cholesterol levels. This suggests that body mass and systemic inflammation form a nexus through which diet and metabolic factors influence cardiovascular risk. This positioning of BMI and CRP as hubs complements the summary correlations and clarifies their roles within the broader dependency structure. [37].

#### 4.3.2. Score-Based (Hill-Climbing) Network

The score-based Bayesian network (learned via the Hill-Climbing algorithm) produced a more complex network of relationships, revealing additional connections and interactions among the variables. One key insight from this model was the strong influence of allostatic load as an upstream factor. In the Hill-Climbing network, allostatic load emerged as a major precursor to several outcomes: it had directed links to blood pressure, triglycerides, and even BMI. This suggests that chronic stress may be driving both metabolic changes (like weight gain and high triglycerides) and vascular changes (hypertension) in our data. Such a result aligns well with clinical understanding of stress physiology: chronic stress responses (e.g., repeated cortisol surges) can promote central adiposity, insulin resistance, and sympathetic overactivity, thereby elevating triglycerides and blood pressure over time [38].

The Hill-Climbing network also indicated more extensive involvement of environmental variables. Compared to the constraint-based model, the score-based approach more directly connected toxic exposures and lifestyle factors with clinical outcomes. For example, the model found that certain environmental exposures (such as lead or cadmium) and behaviors (smoking, diet) were linked not only indirectly through demographic factors but also had direct associations with outcomes, including cholesterol and blood pressure. This suggests that the algorithm detected subtle influences of environmental toxicants on physiological measures that the constraint-based method did not explicitly show [39].

The Grow-Shrink network also highlighted how environmental and social factors interface with health outcomes. It showed robust connections between toxic environmental exposures and sociodemographic variables. For example, nodes related to lead and perfluorinated compounds (PFAS) were linked to factors such as income, education, age, and gender in the network. This pattern suggests that exposure to harmful substances is not random; rather, it is structured by social determinants, an observation consistent with the concept of environmental injustice. In other words, the model reflects that certain groups (e.g., lower-income or less-educated individuals) may bear higher burdens of exposure to toxicants, which can in turn impact their cardiovascular health [40]. This result is consistent with research indicating that social disparities lead to unequal exposure to cardiovascular risks, including toxins and pollution. Additionally, the constraint-based model underscored the influence of diet and stress on cardiovascular physiology. Notably, the DII and allostatic load were connected to blood pressure in the network. This indicates that a pro-inflammatory diet and high chronic stress have direct associations with hypertension in our data. Such a relationship aligns with established causal pathways: diets high in processed, high-fat foods can raise blood pressure, and chronic stress can dysregulate neuroendocrine responses, leading to sustained blood pressure elevation [41].

#### 4.3.3. Strengths of the Network Analysis

This study employed two complementary structure-learning algorithms—Grow-Shrink (constraint-based) and Hill-Climbing (score-based). The use of both approaches enhanced methodological rigor by enabling cross-validation and reducing the potential for bias inherent in any single algorithm. Links consistently identified by both methods were interpreted as more robust and reliable, whereas discrepancies between methods were explicitly acknowledged and considered exploratory, thereby providing readers with a transparent account of analytical uncertainty. In addition, the directed acyclic graph framework offers intuitive interpretability. By delineating Markov blankets and conditional independences, the analysis identifies variables that are directly connected to cardiovascular indicators as well as those that exert influence indirectly through mediating pathways. This capacity to distinguish proximal from distal determinants highlights potentially modifiable factors—such as BMI, CRP, and allostatic load—that may represent key targets for intervention. The Bayesian network framework can model social, environmental, and behavioral factors at the same time, so the results reflect real-world co-exposures and upstream social conditions rather than single-exposure analyses. Finally, the Spearman correlation matrix provided a simple check of expected patterns, for example the link between LDL and total cholesterol, which supports the face validity of the learned networks and makes the findings easier to use for public health decisions.

### 4.4. Comparing Algorithms and Network Structures

Comparing the constraint-based (GS) and score-based (HC) Bayesian network models reveals that they offer complementary perspectives on the same system. The Grow-Shrink model, by design, was more conservative. It included fewer connections, focusing on those relationships strongly supported by the data. In doing so, it highlighted the core drivers of the system: variables like BMI, CRP, diet, and stress emerged as fundamental, and only the clearest pathways among them were retained. This parsimony suggests that the GS network is likely to show the most reliable associations (i.e., those least likely to be statistical artifacts). For example, both models identified obesity and inflammation as central factors; however, the GS model distilled the network to emphasize that BMI and CRP influence many other nodes, with fewer secondary links. One could liken the GS model to a high-specificity approach: it may miss some weaker associations, but the ones it shows are credible and actionable [42].

In contrast, the Hill-Climbing model presented a denser network with a larger number of edges. It captured additional plausible links, effectively providing a high-sensitivity view of the system. Some of these extra connections may represent genuine but subtle relationships (for instance, minor pathways or interactions that exist in reality), while others might be spurious or artifacts of the algorithm’s attempt to improve the overall score. The HC model’s inclusion of allostatic load as a predictor of multiple outcomes, along with the direct ties between more distal factors (such as environmental exposures) and health metrics, illustrates its more exploratory nature [43]. It tends to be sensitive to complex interdependencies for example, it might connect an exposure to an outcome even if that link operates only under certain conditions or through multiple intermediate steps [43]. Despite their differences, both models agreed on several fundamental structures, notably the significance of BMI and CRP, and the interplay of diet, stress, and classical risk factors [44]. The results of the GS model may be easier to understand from the perspective of causality and intervention, as they indicate a more straightforward chain of effects that can be targeted. Taken together, the GS model emphasized high-specificity edges that persisted under resampling, while the HC model captured additional, lower-stability links consistent with higher sensitivity. This pattern is expected, given the algorithm design, and supports reading shared edges as more robust associations in this cohort.

### 4.5. Social and Environmental Determinants of Health

Both Bayesian network models and the underlying data underscored that cardiovascular risk is not merely a matter of personal health metrics but is deeply embedded in social and environmental contexts. A clear theme that emerged is the role of social determinant factors like socioeconomic status (SES), education, race, and gender in shaping exposure and vulnerability. For instance, our analysis found that lower income and education were associated with higher levels of toxic exposures (like lead, mercury, PFAS), as well as with higher allostatic load [45]. This reflects real-world patterns: economically disadvantaged communities often face greater environmental risks (e.g., industrial pollutants, substandard housing with lead paint) and higher chronic stress, contributing to worse health outcomes [46].

These findings align with the broader literature on health disparities, which demonstrates that social and economic adversity contribute to elevated cardiovascular risk via multiple interrelated pathways. For instance, residents of lower-income communities may experience limited access to nutritious food and safe environments for physical activity, heightened psychosocial stress due to financial insecurity or discrimination, and increased exposure to environmental contaminants [47]. Demographic variables such as race and gender also emerged as influential nodes within the network models, suggesting they modulate the distribution of cardiovascular risk factors. The constraint-based Bayesian network further revealed associations between lead and cadmium exposure with both gender and smoking status, highlighting the role of behavioral and sociodemographic determinants in shaping toxicant burden [48]. Observed links between lower socioeconomic position and higher toxicant markers align with environmental-justice literature; however, exclusion of incomplete cases may under- or over-represent some subgroups. As a result, population-level gradients should be interpreted as indicative rather than definitive and warrant validation in larger representative datasets.

### 4.6. Public Health and Policy Implications

The integrative findings from this analysis suggest several avenues for public health action and policy. First, the identification of obesity and inflammation (BMI and CRP) as central, modifiable risk factors points to these as primary intervention targets. Public health programs should continue to aggressively promote healthy weight through nutrition and physical activity, as well as clinical interventions for weight loss, given the likely ripple effects on blood pressure, lipids, and inflammation [49]. Our results indicate that reducing obesity would attenuate systemic inflammation and favorably influence multiple downstream CVD risk factors. This aligns with evidence that weight loss can lower CRP and improve blood pressure and cholesterol levels, thereby reducing the overall risk of heart disease [50].

The findings will help design specific strategies to lower the risk of CVD, especially among vulnerable groups who face environmental and socioeconomic challenges. These populations may include low-income communities, people living in polluted areas, and those with limited access to healthcare. Understanding how these factors contribute to higher CVD risks allows for the creation of more targeted public health programs [51]. Such programs can address the unique needs of at-risk groups, ultimately reducing health disparities and improving well-being for those most affected by CVD. Early-life adversities, such as exposure to pollution, socioeconomic stress, and limited healthcare access, significantly increase the risk of developing CVD later in life. Children who face these stressors are more likely to experience poor cardiovascular outcomes as adults. This highlights the need for interventions that begin early and address these issues before they compound over time [52].

Social factors, such as income, education, and access to healthcare, also play a crucial role in shaping health outcomes. Communities with fewer resources often lack access to preventive healthcare and live in environments that increase their exposure to harmful factors like air pollution and poor nutrition. These conditions, along with limited social support, place them at a higher risk for cardiovascular issues [53].

Additionally, exposure to secondhand smoke during childhood has long-term cardiovascular consequences, with studies showing that children in lower-income and minority communities are disproportionately affected. Secondhand smoke exposure increases the risk of CVD, and those from disadvantaged backgrounds face a greater burden due to factors such as limited access to healthcare and higher rates of tobacco use in their communities [54]. These findings further highlight the importance of addressing environmental and social challenges in the effort to reduce CVD risk [55]. By considering these factors, public health strategies can be tailored to meet the needs of vulnerable populations. This approach not only targets the root causes of CVD but also ensures that resources are directed toward the groups that need them most, ultimately contributing to greater health equity.

Bayesian Networks have become an important tool in epidemiological research, helping researchers better understand the factors that influence disease spread and outcomes. BNs are particularly effective in exploring genetic epidemiology data, allowing for the modeling of complex relationships between genes, environmental factors, and disease risks as shown by [56].

BNs are especially useful for studying diseases with multiple risk factors, as they can handle the uncertainty and complexity involved in understanding how these factors interact. For example, BNs can analyze how lifestyle, genetics, and environmental exposures contribute to disease development, helping to identify key risk factors [57]. By considering different environmental exposures and their possible impacts on human health, BNs can improve environmental health risk assessments. Because of their capacity to combine data from various sources, BNs are a flexible and effective tool for improving public health forecasts and decision-making.

### 4.7. Limitations and Future Directions

This study utilized observational data, which limits the confidence with which we can draw conclusions about cause and effect. While Bayesian networks help identify patterns and associations, they do not confirm that one factor directly causes another. For instance, the model shows a connection between C-reactive protein (CRP) and high blood pressure. However, this does not mean that CRP causes elevated blood pressure. Other factors, such as stress or undiagnosed conditions, may influence both variables.

Another limitation lies in the sample size. After removing incomplete records, the dataset included 601 participants. This was enough to train and analyze the model. Still, it may not reflect the larger population or diverse communities. Results drawn from this group might not apply to others, especially those from different regions, age groups, or health backgrounds. Future studies should use larger and more varied datasets to ensure that the findings are widely applicable and representative. Complete-case analysis can bias network estimates if missingness is not completely at random; therefore, generalization should be made with caution. Future work should include assessing stability with bootstrapped structure learning and comparing results after multiple imputations of missing covariates.

Another limitation is that our analysis focused only on metals and PFAS, as these were the exposure biomarkers available in NHANES 2017–2018. Other environmental factors, particularly air pollution, were not included but are known to influence stress and inflammatory pathways relevant to allostatic load. For example, particulate matter (PM10 and PM2.5) has been associated with alterations in cortisol and TNF-α, suggesting potential contributions to systemic inflammation and dysregulation of stress response systems [58]. Future studies integrating air pollution with chemical exposures could provide a more comprehensive understanding of environmental influences on cardiovascular risk.

## 5. Conclusions

This study highlights the value of Bayesian network modeling in providing a more integrative view of cardiovascular risk. By incorporating biomarkers of inflammation, environmental toxicant exposures, and social factors such as income and education, this approach moves beyond traditional methods that assess risk factors in isolation. Bayesian networks reveal interdependencies among biological, behavioral, and contextual variables; for example, how psychosocial stress and poor diet may elevate inflammation, influencing blood pressure and lipid levels. Socioeconomic disadvantage can also heighten vulnerability to environmental exposures, compounding risk.

The resulting network structure uncovers key pathways through which social, behavioral, and environmental factors intersect to shape cardiovascular health, reflecting the complex, multifactorial nature of chronic disease. This systems-level approach is critical for designing effective prevention and intervention strategies. Tackling cardiovascular disease requires not only clinical treatment but also upstream efforts that address structural and social determinants.

## Figures and Tables

**Table 1:** Description of variables in the study dataset.

Variable Type	Variables	Description
Outcome Variables	Diastolic Blood Pressure, Systolic Blood Pressure, Total Cholesterol, LDL Cholesterol, Triglycerides, HDL Cholesterol	Key cardiovascular health indicators
Health Predictors	Allostatic Load, Dietary Inflammatory Index (DII), body mass index (BMI), C-reactive protein (CRP)	Measures of stress, inflammation, and metabolic health
Environmental Predictors	PFOA, PFOS, Lead, Cadmium, Mercury	Exposure to toxic substances
Lifestyle Predictors	Alcohol, Smoking	Behavioral risk factors
Socioeconomic Predictors	Education, Income	Social determinants of health
Demographic Predictors	Age, Gender, Race	

**Table 2:** Descriptive statistics for key health variables.

Variable	25th Percentile	Median	Mean	75th Percentile
Allostatic Load	3.00	3.00	3.59	5.00
DII (Dietary Inflammation Index)	0.35	1.64	1.49	2.74
Diastolic Blood Pressure	64.67	72.67	72.67	79.33
Systolic Blood Pressure	112.67	123.33	125.96	136.67
Total Cholesterol	155.00	182.00	185.10	209.00
LDL Cholesterol	85.00	106.00	109.90	129.00
Triglycerides	59.00	89.00	104.40	130.00
HDL Cholesterol	42.00	51.00	54.39	62.00
BMI	24.50	28.20	29.81	34.00
CRP	0.80	1.79	4.43	6.09

**Table 3:** Summary of Bayesian network structure learning parameters using the grow-shrink algorithm.

Parameter	Value	Explanation
Nodes	22	Number of variables (e.g., BMI, Blood Pressure, etc.) in the model
Total arcs	44	Total number of edges (connections) in the network
Directed Arcs	32	Edges with a defined direction, suggesting potential causal influence
Undirected Arcs	12	Edges with no assigned direction, indicating potential association only
Average Markov Blanket Size	5.00	Average number of variables in each node’s Markov blanket
Average Neighborhood Size	4.00	Average number of adjacent (connected) nodes per variable
Average Branching Factor	1.45	Average number of outgoing edges per node
Conditional Independence Test	Pearson’s Correlation	Statistical test used to check if two variables are related
Alpha Threshold	0.05	Significance level used in independence testing
Tests Used in Procedure	3917	Total number of statistical tests conducted during network structure learning

**Table 4:** Summary of Bayesian network parameters (Score-Based Method).

Parameter	Value	Explanation
Nodes	22	Total number of variables included in the network
Total Arcs	44	Number of edges (relationships) identified between variables
Directed Arcs	44	All arcs have assigned direction, indicating potential causal influence
Undirected Arcs	0	No undirected arcs present in the final model
Average Markov Blanket Size	6.09	Average number of variables in each node’s Markov blanket
Average Neighborhood Size	4.00	Average number of directly connected neighbors per variable
Average Branching Factor	2.00	Average number of outgoing arcs per node
Scoring Function	Bayesian Gaussian (BGe)	Scoring metric used to evaluate model fit
Imaginary Sample Size (Normal)	1	Prior parameter controlling strength of the normal component
Imaginary Sample Size (Wishart)	4	Prior parameter for the Wishart distribution in the BGe score
Test used in procedure	1281	Total number of model comparisons evaluated during structure learning
Optimized	True	Indicates the algorithm successfully identified a high-scoring network model

## Data Availability

The data presented in this study are openly available on the CDC NHANES site at https://wwwn.cdc.gov/nchs/nhanes/continuousnhanes/overview.aspx?BeginYear=2017 (accessed on 1 July 2024).
